# Evaluation of lived experience Peer Support intervention for mental health service consumers in Primary Care (PS-PC): study protocol for a stepped-wedge cluster randomised controlled trial

**DOI:** 10.1186/s13063-024-08165-y

**Published:** 2024-05-14

**Authors:** Sharon Lawn, Tania Shelby-James, Sam Manger, Louise Byrne, Belinda Fuss, Vivian Isaac, Billingsley Kaambwa, Shahid Ullah, Megan Rattray, Bill Gye, Christine Kaine, Caroline Phegan, Geoff Harris, Paul Worley

**Affiliations:** 1 College of Medicine and Public Health, Flinders University, GPO Box 2100, Adelaide, SA 5001 Australia; 2 Lifestyle Medicine, James Cook University, James Cook University LPO, 150 Angus Smith Drive, Douglas, QLD 4814 Australia; 3 School of Management, RMIT, GPO Box 2476, Melbourne, VIC 3001 Australia; 4 Faculty of Science and Health, Charles Sturt University, Albury, NSW 2640 Australia; 5 Community Mental Health Australia, PO Box 668, Rozelle, NSW 2039 Australia; 6 Lived Experience Australia, PO Box 96, 5048 Brighton, Australia; 7 Mental Health Coalition of South Australia, Suite 2/195 North Terrace, Adelaide, SA 5000 Australia

**Keywords:** Mental health, Primary care, Mental health services, Consumers, Peer workers, Lived experience workforce, Cluster-randomised trial

## Abstract

**Background:**

The demand for mental health services in Australia is substantial and has grown beyond the capacity of the current workforce. As a result, it is currently difficult for many to access secondary healthcare providers. Within the secondary healthcare sector, however, peer workers who have lived experience of managing mental health conditions have been increasingly employed to intentionally use their journey of recovery in supporting others living with mental health conditions and their communities. Currently, the presence of peer workers in primary care has been limited, despite the potential benefits of providing supports in conjunction with GPs and secondary healthcare providers.

**Methods:**

This stepped-wedge cluster randomised controlled trial (RCT) aims to evaluate a lived experience peer support intervention for accessing mental health care in primary care (PS-PC). Four medical practices across Australia will be randomly allocated to switch from control to intervention, until all practices are delivering the PS-PC intervention. The study will enrol 66 patients at each practice (total sample size of 264). Over a period of 3–4 months, 12 h of practical and emotional support provided by lived experience peer workers will be available to participants. Scale-based questionnaires will inform intervention efficacy in terms of mental health outcomes (e.g., self-efficacy) and other health outcomes (e.g., healthcare-related costs) over four time points. Other perspectives will be explored through scales completed by approximately 150 family members or carers (carer burden) and 16 peer workers (self-efficacy) pre- and post-intervention, and 20 medical practice staff members (attitudes toward peer workers) at the end of each study site’s involvement in the intervention. Interviews (*n* = 60) and six focus groups held toward the end of each study site’s involvement will further explore the views of participants, family members or carers, peer workers, and practice staff to better understand the efficacy and acceptability of the intervention.

**Discussion:**

This mixed-methods, multi-centre, stepped-wedge controlled study will be the first to evaluate the implementation of peer workers in the primary care mental health care sector.

**Trial registration:**

Australian New Zealand Clinical Trials Registry (ANZCTR) ACTRN12623001189617. Registered on 17 November 2023, https://www.anzctr.org.au/Trial/Registration/TrialReview.aspx?id=386715

**Supplementary Information:**

The online version contains supplementary material available at 10.1186/s13063-024-08165-y.

## Introduction

Mental health conditions account for 12% of fatal and non-fatal health burden in Australia, having the second highest non-fatal health burden after musculoskeletal disability [1]. A substantial number of Australians—21.5% of those aged 16–85 experienced difficulties in the past 12-month period or 42.9% across their lifespan [2]. Despite the mental health workforce capacity increasing by 6.5% in the period 2021–2022, availability has been surpassed by growing demand for mental health services [3]. This is especially so for priority groups (e.g., rural and remote) due to social determinants, geographical barriers, costs, and an even further reduced workforce capacity for providing services [3]. Difficulties accessing services has been further exacerbated by significant adverse events such as climate disasters and the COVID-19 pandemic [4, 5]. Economic costs associated with the figures are estimated to be between $43 and 70 billion per year [3]. Reports indicate that the current mental healthcare system requires wider reforms to provide equitable access to adequate support for the diverse needs of people living with mental health conditions [3, 6].

Australia has long emphasised a recovery-orientated approach in national and state/territory mental health plans and policy, aiming to go beyond an ‘illness’ model [7, 8]. This aligns with the desires of those living with mental health conditions to access mental health services earlier and to have more control over their own health [3, 8, 9]. Primary care services, including general practices and psychological services, play a pivotal role in offering this support and making referrals to specialised support [9]. However, many Australians face significant wait times and limited access (e.g., geographical, financial) to these services. This has led a considerable number of people seeking help at emergency departments in crisis or to slip through service gaps in a system increasingly focused on crisis management [3, 8, 10]. Therefore, there is an urgent need to enhance the delivery and quality of recovery-oriented mental health support through recovery-focused primary care interventions that give people living with mental health conditions a greater say in what supports are offered.

As a crucial step toward person-focused recovery-orientated care, peer support delivered by peer workers is increasingly being trialled worldwide [11–13]. Peer workers have lived experience (LE) of managing mental health conditions and are intentionally employed to leverage their experience of recovery to assist and support people living with mental health conditions and their network of informal supports [14]. Interaction between peer workers and others living with mental health conditions is founded on mutuality, equality, and reciprocity [13], with peer workers metaphorically ‘walking alongside’ the person living with mental health conditions whilst sharing their own recovery experience [7]. Further, peer workers connect people living with mental health conditions to services and community activities, aiding in customising services to their individual needs. LE peer support is multifaceted, involving positive self-disclosure, expansion of social networks, education, and advocacy. Ultimately, peer workers foster trust and engagement with services amongst people living with mental health conditions, concurrently reinforcing self-efficacy, connectedness, and resilience [7, 13].

Currently, peer workers are employed in various secondary and tertiary settings [13, 15], including emergency departments [16], crisis management [17], and community services [4]. A pilot study demonstrated the acceptability, feasibility, and effectiveness of peer support in preventing hospital admissions, lowering re-presentations, and reducing costs. This outcome led to the funding of a successful state-wide peer-supported post-hospital community service in Australia [18, 19]. Further, international randomised controlled trials (RCTs) [20, 21] and systematic reviews [22, 23] have confirmed the effectiveness of peer support in acute care settings through demonstrating improved psychosocial and care outcomes. Despite existing evidence suggesting that peer support yields more responsive, safe, effective, and person-centred care [22], peer workers remain absent from the primary care workforce in Australia and worldwide.

General practitioners (GPs) are vital to primary mental health care as they act as the principal referrers for supports. Approximately 5 million Australians (25%), including 2.3 million with mild and 1.1 million living with moderately severe mental health conditions, seek support from GPs annually [24]. This level of engagement suggests an opportunity to supply additional supports at the primary care level which are available, accessible, and recovery focused. To address the challenges surrounding the provision of mental health care in the primary setting, we are trialling the implementation of a lived experience Peer Support intervention for those accessing mental health care in Primary Care (PS-PC). The PS-PC intervention will be offered in four general practices—three regional and one remote—with the intention to empower those living with mental health conditions to improve their self-efficacy, personal recovery, access to services, and quality of life.

## Objectives

The primary objective of this study is to understand the efficacy of the PS-PC in enhancing the self-efficacy (i.e., a person’s belief in their ability to manage their mental health and recovery) of those who are accessing mental health services at a primary care level (i.e., mental health service consumers). A secondary objective is to assess the feasibility of peer worker support in the general practice setting and identify barriers to implementation.

## Methods

### Study design and setting

This mixed-method, multi-site, stepped-wedge cluster RCT trial will evaluate the PS-PC in the primary care setting across four Australian practices (study sites). The PS-PC intervention was designed using the Experience Based Co-Design (EBCD) Framework, with a focus on the combined experiences and knowledge of those who provide and receive mental healthcare [25]. The experiences of the providers and recipients of care were considered in the co-design process through:

- A Project Reference Group (PRG) consisting of all investigators involved in the project. The PRG is responsible for monitoring the research activities within the project, including the management of challenges at the project management level.
- A Stakeholder Reference Group (SRG) consisting of individuals or organisations in the community that have a stake in the outcomes of the PS-PC project, including mental healthcare consumers. The SRG is responsible for advising and overseeing all project activities to ensure that the experience of community stakeholders was included across the project.
- Semi-structured interviews held with Australian mental healthcare consumers, family/carers, and peer workers to understand the experiences of those engaged with mental health care services.
- Three workshops (one at each study site 1–3), held with practice staff, mental healthcare consumers from the community, sector specialists, peer workers, and CMO representatives. Workshops were held on-site, but virtual attendance via Microsoft Teams was available to be inclusive for all invitees. Workshops were intended to foster relationships between different groups, to introduce the peer role to those who were unfamiliar, and to understand how to best tailor the intervention to each study site.
- Semi-structured interviews were held with GPs, practice managers, nurses, and reception staff at study sites 1–3 to understand the experiences of those who provide mental healthcare services at the primary care level and how peer workers might be accommodated in this space.

Guidance in constructing the intervention came from these sources through thematic analysis (deductive and inductive), engagement with the SRG and PRG, and ongoing communication between the research officer, practice managers, and peer coordinators. See Additional File 1 for more detail.

**Fig. 1:**
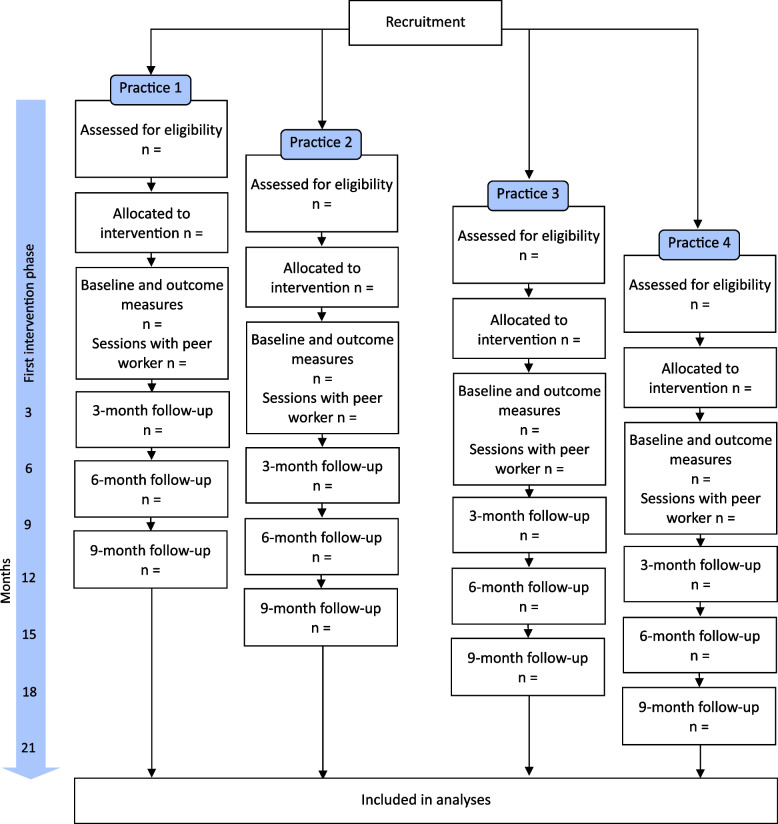
Study diagram (based on CONSORT [27]) for flow of participants by study site

Design and selection of planned evaluation methods followed the Consolidated Framework for Implementation Research (CFIR) [26] and RE-AIM (Reach, Effectiveness, Adoption, Implementation, Maintenance) Framework. A study diagram based on CONSORT demonstrating the planned reporting of the intervention can be seen in Fig. 1.

The study will take place in four general practice clinics across rural and regional areas of Australia. Peer workers will be employed by community-managed organisations (CMOs) that are able to provide services to these areas. The locations of participating sites is available on the trial registration page [28]. Approval for the study has been granted by the Flinders University Human Research Ethics Committee (#6386). The SPIRIT reporting guidelines (see Additional File 2) were used in the development of this protocol [27].

## Sample size

The sample size calculation (*n* = 264 participants; 66 per practice) is based on the primary outcome of the self-efficacy score from an RCT assessing the effectiveness of peer support for mental health outpatients in Germany [29]. Adjusted scores significantly improved by an average of 1.77 points (95% CI 0.02–3.53) in the peer support group compared to usual care (mean score = 24.3, pooled SD = 7.2) in the 6-month follow-up. As this study is based on 9-month follow-up, the upper confidence level of the improved score will be used. Assuming a 10% attrition rate, we will need to recruit 66 participants per practice with a total of 264 participants. This achieves at least 80% power to detect a difference of mean self-efficacy score of 3.53 points. A power and sample-size analysis for hypothesis test from Stata version 16.1 was used to calculate the sample size*.* The test statistic used is the one-sided Wald *Z*-test and the significance level of the test is 0.05. Assuming heterogeneity amongst practices and substantial differences (i.e., minimal resemblance) in participants at each site, the responses of participants will not be significantly influenced by their enrolment at a particular practice. As such, the ICC is 0.01.

## Randomisation

The four practices will be randomly allocated by the study statistician to receive the standardised PS-PC intervention until all four sites have implemented it. Timing of allocation will depend on the relative needs of the practices and peer worker providers (e.g., staffing, leave arrangements, time of year). At the initialisation of each wedge, staff leaders at the relevant practice site will champion the practical implementation of the PS-PC, beginning with recruitment strategies. All participants will receive usual care, plus the addition of peer support when their study site switches to the PS-PC intervention. Due to the trial’s specific nature, neither randomisation nor blinding of participants will take place. Participants have the freedom to withdraw from the intervention at any time upon their request.

## Recruitment and enrolment

GPs at each enrolled practice will use prospective enrolment and compile a list of people living with mental health conditions currently attending the practice who may benefit from the PS-PC intervention. GPs will introduce the study to potential participants and provide key details. The study will also be promoted within the waiting rooms of participating practices for people living with mental health conditions to self-nominate for eligibility screening. GPs will then screen participants for eligibility and those wanting to participate will be referred to a designated practice nurse to complete informed consent processes. Information about those who choose not to participate at this point will be documented with consent.

## Eligibility criteria

A person will be eligible to participate if they are as follows:

- 18 years of age or older
- Have a diagnosis of a mental health condition (or present with symptoms indicating the likelihood of a mental health condition)
- Attend participating practices (study sites)

A person will be ineligible to participate if they:

- Have a significant mental health condition or physical illness likely to disrupt their capacity to participate in the trial (as measured by GP assessment of current suicidality risk and/or Kessler psychological distress scale (K10) cut of score of > 40/50)
- Are unable to speak English
- Are already receiving peer support through an alternative funded programme

To gain greater understanding of the impact of the trial beyond the participant, a subset of family members or carers will complete the short-form Burden Scale for Family Caregivers (BSFC-s) [30]. Participants who are willing and have a suitable person to nominate will be provided with a consent form, questionnaire, and reply paid envelope to give to a family member or carer. Family members and carers can opt in to be part of the study at this point by either returning the completed forms or following a link to an online version. No inclusion or exclusion criteria apply beyond being nominated by the participant.

Peer workers involved in the study will complete the General Self-Efficacy scale (GSE) to inform intervention efficacy for those providing care. Additionally, a scale will be developed to measure evaluate participating practice staff attitudes toward peer workers. This scale will be informed by the components involved in the Theoretical Framework of Acceptability (TFA) [31]. Peer worker and practice staff participants will be recruited through their employer, with no obligation to be involved and no requirements for inclusion beyond their employment. These participants will be informed about the data collection component of their involvement in the study and will give written consent to participate when completing the forms. Completed forms can be provided to study staff via the study contact representative at their workplace (either the peer coordinator or practice manager) or by following a link to an online version.

## Intervention

**Fig. 2:**
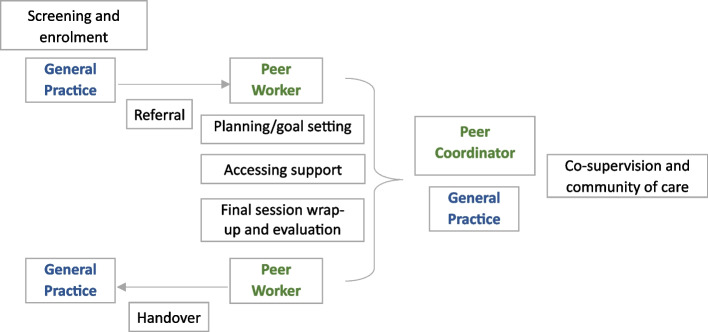
Intervention stages and flow of care

Each participant allocated to the intervention will be matched by a ‘peer coordinator’ (a champion peer worker coordinating between peer workers and the study staff) to the skills and experience of a peer worker. The main intervention steps and the flow of health care providers can be seen in Fig. 2.

The initial meeting between the participant and peer worker will provide an opportunity to refine and mutually agree on the support plan. Support may entail emotional and practical assistance, phone calls, home visits, community linkage, and sharing of strategies to enhance lifestyle behaviour (e.g., social connection, physical activity, stress management) to build self-efficacy and self-care and to set recovery goals.

Peer workers will provide 8–12 h of support per participant over approximately 2–3 months, with the option to extend for 2–3 months if needed. If desired, participants may also cease receiving peer worker support sessions prematurely, if they feel they have gained sufficient benefit from these sessions. At the completion of the intervention, the peer worker and participant will identify supports that are relevant and available and the peer worker will link the participant with those supports. The peer worker will provide a summary of trial activities to the participant and their GP. This is intended to be used for discussion in a primary care appointment to facilitate ongoing continuity of care.

## Data collection

**Fig. 3:**
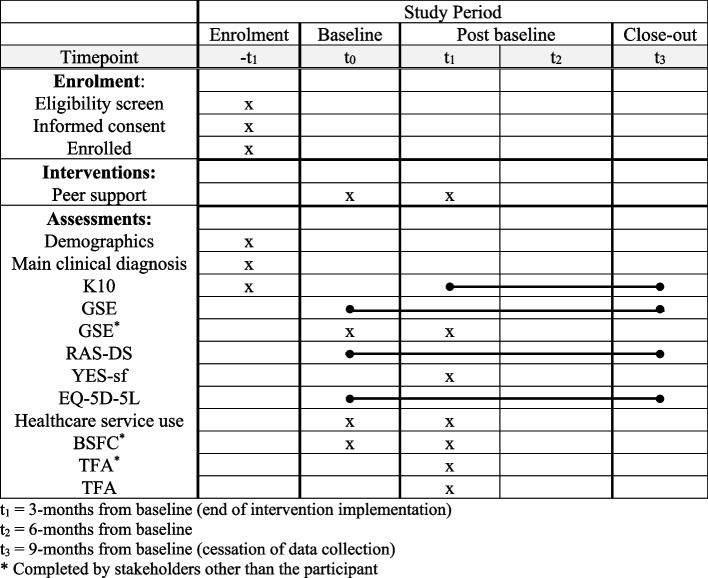
Schedule of enrolment, intervention, and assessment (SPIRIT Figure)

A timeline for the collection of data can be seen in Fig. 3.

A summary of the evaluation schedule, underpinned by the RE-AIM framework is provided in Table 1. Routinely collected data including demographics and diagnosis details will be recorded (with consent) by practice staff through chart auditing the GP records of prospective participants. At baseline and during the final peer support session, participants will have the option of completing data collection forms on paper, using iPads available at each study site or using another digital device. Data from paper forms will be entered into the REDCap database by the practice manager, practice nurse, or research officer. Paper data collection forms will be audited against online data at the end of the study for accuracy and completeness. All data collection forms are available on request.

**Table 1:** Evaluation schedule with RE-AIM framework domains

RE-AIM Domain	Measure	Evaluation process
Reach	Number of people living with mental health conditions referred to the project	Analysis of routinely collected process data
	Characteristics (i.e., age, gender, diagnosis, treatment) of those who consent to participate vs. those who did not	
Effectiveness		
*Primary*	Participant self-efficacy (GSE)	Collected at enrolment (-t_1_) or baseline (t_0_) 3, 6, 9 months (t_1_ to t_3_) post-baseline
*Secondary*	Patient psychological distress (K10) Participant personal recovery (RAS-DS) Cost-effectiveness (EQ-5D-5L)	
	Family member or carer burden (BSFC-s) Peer worker self-efficacy (GSE)	Collected pre- (t_0_) and post-intervention (t_1_)
	Participant satisfaction with service (YES^a^) Practice staff attitudes towards peer workers^b^ Participant service use costs^c^ Perceived effectiveness of trial^b^	Collected post-intervention (t_1_) only
Adoption	Perspective of participants, family members and carers, peer workers, and practice staff	Semi-structured interviews and focus groups Analysis of routinely collected process data
Implementation	Frequency and content of participant-peer worker encounters	
Maintenance	Feedback from participants, family members and carers, peer workers, and practice staff	

^a^Short-form adjusted for CMOs

^b^Measure (Likert scale) developed for the study, in (TFA)

^c^Self-report questionnaire of service use and associated costs

## Primary outcome measures

The primary outcome will be change in a person’s self-efficacy measured by the GSE [32]. The GSE is a reliable (*α* = 0.76 to 0.90), validated, ten-item unidimensional self-report scale used to assess self-efficacy within general adult populations. The scale conceptualises self-efficacy as a person’s perceived ability to cope and adapt in the face of both daily hassles and more stressful life events. Respondents indicate how much a given item applies to them from 1 (not at all true) to 4 (exactly true), with total possible scores of 10–40 (higher scores indicate greater self-efficacy). The scale was originally designed to assess change occurring over the course of a trial and is also used in CMO contexts as an indicator of client well-being. This tool will be used to inform intervention efficacy for participants at baseline, 3 months post-baseline, 6 months post-baseline, and 9 months post baseline. For all measures taken at multiple time points, the comparison between care as usual (baseline) and post-intervention (3 months post-baseline) will be of primary importance, whilst following time points will inform the longevity of any intervention-related effects.

All baseline measures, including the GSE, will be administered as a questionnaire by a practice nurse following screening and consent procedures. During the final session of peer support, the peer worker will assist the participant with completing the 3-month post-baseline measures. All further follow-up measures will be administered as a questionnaire either through REDCap or a telephone call from study staff depending on participant preference.

## Secondary outcome measures

### Efficacy

1. Psychological distress: The Kessler Psychological Distress Scale (K10) is a reliable (*α* = 0.93), validated, 10-item unidimensional self-report measure of participant mental distress [33] often used by clinicians as a screening tool and marker of eligibility for government-funded support services. Respondents indicate from 1 to 5 how much a given statement was true for them in the past 4 weeks with total possible scores of 10–50 (lower scores indicate less distress). The GP or a study nurse will administer the K10 to potential participants as a screening material to aid in confirming eligibility, and then at 3 months post-baseline, 6 months post-baseline, and 9 months post-baseline.
2. Personal recovery: The Recovery Assessment Scale—Domains and Stages (RAS-DS) [18] is a reliable (*α* = 0.96), validated, 38-item multidimensional self-report measure of personal recovery designed to measure the recovery-oriented impact of services provided to participants. The scale is constructed of five domains relevant to recovery goals, with respondents indicating how much a given statement is true for them from 1 to 4. Higher scores in each domain or stage indicates areas the person feels more confident about as part of their recovery. The RAS-DS is routinely used by peer workers as a descriptive tool. This tool will be used to inform intervention efficacy for participants at baseline, 3 months post-baseline, 6 months post-baseline, and 9 months post-baseline.
3. Satisfaction with service: The Your Experience of Service (YES) is a descriptive short-form (21-item) version of the Australian YES [34] tailored to evaluating CMOs. The YES is used in evaluating services delivered by government and non-government healthcare providers in Australia. Respondents indicate their experiences from 0 to 5 across three areas (higher score indicates more satisfaction with a service). The measure then prompts responses to open-ended questions for further information. This measure will be completed during the participant’s final session.

### Health outcomes and health services utilisation

1. Quality of life years: The European Quality of Life 5-Dimension 5-Level scale (EQ-5D-5L) [35] is a reliable (*α* = 0.82), validated, 5-item multidimensional measure commonly used in the field of health economics to estimate the number of quality-of-life-years resulting from an intervention to predict future costings. Respondents indicate which statement they agree with most regarding their health ‘today’ for five health domains with lower scores indicating better health. Respondents also provide an indicator of their general health on a scale of 1–100 (higher scores indicate better health). This measure will be used to inform the intervention efficacy in terms of prospective healthcare cost-reduction at baseline, 3 months post-baseline, 6 months post-baseline, and 9 months post-baseline.
2. Healthcare service use.
  A 12-item questionnaire developed for this study will be used to collect information about participant health resource use. The questionnaire is specific to the socio-economic status of participants and costs incurred from accessing services and treatments (e.g., service and treatment costs). This measure will be used to provide context to findings around intervention efficacy in terms of healthcare costs at baseline, 3 months post-baseline, 6 months post-baseline, and 9 months post baseline.
3. Burden experienced by family members or carers: The Burden Scale for Family Caregivers (BSFC-s) [36, 37] is a widely used reliable (*α* = 0.92), validated, ten-item unidimensional self-report measure of subjective carer burden amongst family caregivers. Responders indicate agreement with statements on a scale from 0 (strongly disagree) to 3 (strongly agree), with total possible scores of 0–30 (lower scores indicate lower carer burden). This measure will be used to inform intervention efficacy in terms of ongoing effects for participating family members or carers at baseline, 3 months post-baseline, 6 months post-baseline, and 9 months post-baseline.

### Evaluation of intervention

Stakeholders from a range of perspectives (participants, peer workers, family members or carers, and practice staff) will be invited to take part in evaluating the intervention in terms of efficacy and acceptability.

1. Effectiveness and acceptability.
  Perceived effectiveness of intervention will be measured using a scale adjusted for this purpose. This scale will follow the generic form of the TFA acceptability questionnaire, which is an eight-item theory-informed self-report measure of the acceptability of healthcare interventions [31]. Constructs consider affective attitudes, burden, ethical consequences, perceived effectiveness, coherence, self-efficacy, opportunity costs, and overall acceptability of an intervention. Responders indicate agreement with statements on a scale from 1 to 5, with total possible scores of 8–40 (higher scores indicate higher acceptability). This measure will be completed during the participant’s final session.
2. Attitude toward peer workers.
  Practice staff attitude toward peer workers will be measured using the affective attitude item from the generic form of TFA acceptability questionnaire, which asks either ‘Did you like or dislike [intervention]?’ (participants indicate from 1 (strongly dislike) to 5 (strongly like)) or ‘How comfortable did you feel [behaviour e.g., to engage with] [intervention]?’ (participants indicate from 1 (very uncomfortable) to 5 (very comfortable)) where [intervention] refers to peer workers [31]. This measure will be completed after the final participant has completed the intervention.
3. Qualitative assessment.
  Approximately 6 months after the PS-PC has launched, all participants will be invited via email to take part in interviews of approximately 30 min in length. Peer workers and practice staff will also have the option of participating in a focus group of approximately 1.5 h. Interviews and focus groups will follow a guide developed in reference to CFIR and RE-AIM frameworks. Verbal informed consent will be obtained by the administering study staff member prior to the start of each interview and focus group.

## Retention and withdrawal

To enhance participant retention in the study, the research team will provide convenient and flexible scheduling with easily accessible study locations. Regular check-ins will also be conducted.

All participants will be free to withdraw from the study at any time, for any reason without affecting their usual care or work arrangements (peer workers). Multiple ways to communicate this intent to withdraw will be provided, and processes will be made available, for the participant’s preference. Where relevant, the withdrawing participant will be offered a wrap-up session with their peer worker or GP.

Participants may be withdrawn from the study due to any adverse event associated with their involvement in the PS-PC sessions, where it appears unsafe to continue or results in a serious adverse event where their condition worsens such that they require treatment or hospitalisation for an acute psychiatric episode or other health condition. The worsening of a co-occurring medical or physical condition will only cause the participant to be withdrawn if this significantly impact the participant’s ability to attend sessions. Withdrawals performed by study staff will require deliberation, especially where a participant experiences a worsening of mental health, as withdrawing the participant would lead to a reduction of support when it may be most needed. Where possible, the severity of these withdrawal reasons will be recorded.

## Data privacy and management

Stringent measures will be implemented to ensure the privacy and confidentiality of the participants. Unique study identifications will be assigned to participants to de-identify all responses across longitudinal data collection. All databases will be protected using password and two-factor authorisation encrypted access systems. Because involvement in the trial is short-term and not overly invasive for each participant, and the known risks associated with this intervention are minimal, a data monitoring committee is not necessary. Likewise, there are no plans for interim analyses or development of stopping guidelines.

All identifiable data (consent forms, etc.) will be de-identified and filed with the study documents during the recruitment period. At completion of the study, all participant forms will be sent to the coordinating site by registered mail, for collation and archiving. All participant files will be reconciled and stored along with all study materials—both hard copy and electronic—consistent with the regulations of the Government of South Australia regarding the retention and disposal of participant records.

## Project oversight

The study will be supervised by PRG and SRG. The PRG will oversee project implementation; its membership includes Chief and Associate Investigators and project staff. SRG membership is via PRG invitation and includes community stakeholders with to ensure their perspectives, especially lived experiences, are integrated during project implementation.

### Monitoring

The PRG will be responsible for monitoring the conduct of the study including all adverse events; they will be guided by a Standard Operating Procedure (SOP) which defines adverse events and study related responses. As this is a pragmatic trial being conducted in primary care, any harms will be non-systematically identified through spontaneously reporting by either the participant or peer worker. Incidents will be escalated to the participant’s treating GP who will perform a clinical assessment of risk. All adverse events will be reported to the Flinders University HREC and included within any reported findings. No independent auditing of trial conduct will be undertaken.

Regular communication between research staff and care providers (GPs, practice managers, peer coordinators, peer workers) will be instrumental in key information being shared with relevant persons. Where amendments to the protocol require discussion (i.e., major changes are necessary), the PRG will be consulted. Key Performance indicators have been developed to track study process to ensure recruitment and follow-up are completed within specified timeframes.

## Statistical methods

Independent sample *t*-test and standard Chi-square test for association with continuity correction will compare participant characteristics during usual care with those during mid- and post-intervention. Multivariate multilevel mixed-effects models will be used to examine changes in outcome measures between pre-intervention baseline and post-intervention follow-up over time (6, 12, and 18 months) including interactions between pre-intervention baseline and post-intervention follow-up over time. The comparison between care as usual (baseline) and post-intervention (3-months post-baseline) will be of primary importance, whilst subsequent time points will inform the longevity of any intervention-related effects. This analytical strategy is selected due to the hierarchical nature of the data, where participants are nested within practices. Linear models will be used for continuous outcomes (i.e., GSE, K10, RAS-DS, YES, EQ-5D-5L, TFA) and logit models for binary outcomes (e.g., demographics, diagnosis, etc.). The two-sided test will be performed for all analyses, 95% confidence interval reported, and level of significance set at *p* < 0.05. All analyses will be performed using Stata software version 16.1 and R version 4.1.1. Responses on the EQ-5D-5L integrated with survival curves will be integrated estimate quality adjusted life years using the quality-adjusted survival analysis method. Participant self-reported service use during usual care will be compared with those during and post-intervention to determine within-trial incremental costs and trial effectiveness. Where there is missing data, intention-to-treat protocol will be followed for all participants who receive peer support, where baseline information is available. If there is a significant amount of missing data (missing at random), multiple imputation will be used.

The CFIR menu will guide coding using a descriptive content-coding approach that is both deductive and inductive [38]. Two researchers will independently code each transcript then compare, with any differences resolved through a third analyst. Ratings of valence and manifestation will also be assigned to codes for rigor, and the ‘coherence’ of the data will support its validity [39]. If it is determined that additional data are required for saturation of themes, further data collection will be conducted. Researchers conducting these phases of analysis will be blinded to the general practice that participants attend [40]. The qualitative study will inform analysis and interpretation of the quantitative study findings.

## Discussion

This multi-centre, stepped-wedge controlled study will provide peer support to people seeking care for mental health conditions within general practice. This will be achieved by connecting participants with peer workers, with the intention of improving access to relevant supports, improved self-efficacy in navigating the mental healthcare system, taking control of their own health needs, mitigating psychological distress, and promoting personal recovery. Aligned with the EBCD ethos, the ongoing engagement with various key stakeholder groups will provide opportunities for continuous improvement, monitoring, and maintenance of the intervention and its outcomes.

The study may also further benefit the family members and carers involved in the participant’s care by reducing the amount of support they provide. In addition, peer workers may experience increased self-efficacy, along with greater understanding of general practice, from providing support through this intervention. Furthermore, practice staff may benefit from a greater understanding and acceptance of the peer worker role through exposure to peer worker care processes. The results of this study will provide greater insight into the role of for peer workers within the primary care setting. Finally, findings around the cost–benefit of the PS-PC will inform economic feasibility of peer workers in primary care settings. There is a compelling need for a comprehensive trial of peer support at the primary level [3]. If positive, the study may provide information to support the inclusion of primary mental health peer workers within the Medicare Benefits Schedule. If negative, this adequately powered study will help to inform practice within CMOs and clinical practice. Overall, this study will provide opportunities to extend the role of peer workers throughout the healthcare system.

## Trial status

Protocol v1, finalised on 16/10/2023. Recruitment began 9/11/2023. Approximate anticipated date of recruitment completion 25/08/2025.

### Supplementary Information

Additional file 1: Table - Details of those participating in project co-design.

Additional file 2: Reporting checklist for protocol of a clinical trial.

## Data Availability

Final trial dataset will be retained by Flinders University in South Australia. Access is limited to the investigators involved in the data management of this study (SL, TS-J, SU, BK, MR, BF) due to ethical restrictions on data sharing and the sensitivity of data collected. A copy of the data management procedures is available on request. Findings will be disseminated through publication. This may include peer-reviewed journals, government white papers, presentations to sector leaders, and summaries provided to community stakeholder groups. Authorship will be decided on a case-by-case basis. Ethical approval has not been obtained to make data available to the public.

## Abbreviations

PS-PC: Lived experience Peer Support intervention for mental health service consumers in Primary Care
LE: Lived experience
RCT: Randomised control trial
GP: General practitioner
EBCD: Experience Based Co-Design
CFIR: Consolidated Framework for Implementation Research
RE-AIM: Reach, Effectiveness, Adoption, Implementation, Maintenance
CMO: Community-managed organisation
K10: Kessler psychological distress scale
GSE: Generalised Self-Efficacy scale
RAS-DS: Recovery Assessment Scale—Domains and Stages scale
YES-sf: Your Experience of Service scale
EQ-5D-5L: European Quality of Life—Five Dimension, Five Level scale
BSFC-s: Burden Scale for Family Caregivers short scale
TFA: Theoretical Framework of Acceptability
PRG: Project Reference Group
SRG: Stakeholder Reference Group
