# Supplementary material for: Evaluation of lived experience Peer Support intervention for mental health service consumers in Primary Care (PS-PC): study protocol for a stepped-wedge cluster randomised controlled trial
Source: Trials. 2024 May 14;25:319. doi: 10.1186/s13063-024-08165-y (PMC11094922; doi:10.1186/s13063-024-08165-y)
Supplement: Supplementary file 1 — Additional file 1: Table - Details of those participating in project co-design. [file 13063_2024_8165_MOESM1_ESM.docx]

**Supplemental Materials**

Table: Details of those participating in project co-design.

| **Co-design phase** | **Strategy/tool** | **Format** | **Participants** | ***n*** |
| --- | --- | --- | --- | --- |
| Setting up the project | Engagement | PRG meetings | PRG meetings | 22 |
|  |  | SRG meetings | SRG meetings | 15 |
|  | Project start-up | Start-up workshops | Practice staff | 15 |
|  |  |  | Mental healthcare consumers | 3 |
|  |  |  | Sector specialists | 4 |
|  |  |  | Peer workers | 2 |
|  |  |  | CMO representatives | 3 |
|  |  |  | **Total^1^** | **25** |
| Gathering the experience | Consumer stories | Semi-structured interviews | Mental healthcare consumers | 15 |
|  |  |  | Family/carers of mental healthcare consumers^2^ | 8 |
|  |  |  | Mental healthcare peer workers^3^ | 12 |
|  |  |  | **Total** | **35** |
|  | Staff experiences | Semi-structured interviews | GPs | 4 |
|  |  |  | Practice managers | 3 |
|  |  |  | Nurses | 4 |
|  |  |  | Reception staff | 3 |
|  |  |  | **Total** | **24** |

^1^ Total unique attendees, not including research staff administering workshops. Some attendees attended more than one workshop.

^2^ All peer workers also identified as mental healthcare consumers and almost all (*n* = 10, 83%) identified as family/carers of others living with mental healthcare difficulties but were distinct in their role as peer workers.
